# The importance of spirituality in supportive care

**DOI:** 10.4103/0973-6131.78181

**Published:** 2011

**Authors:** Giuseppina Messina, Stefania Anania, Claudia Bonomo, Laura Veneroni, Antonietta Andreoli, Francesca Mameli, Chiara Ortolina, Paola De Fabritiis, Maria Gaffuri, Francesco Imbesi, Egidio Moja

**Affiliations:** Psychological Service, Foundation IRCCS Ospedale Maggiore Policlinico, Milan, Italy; 1 School of Medical Psychology, San Paolo Hospital, University of Milan, Italy

**Keywords:** Cancer, spirituality, yoga

## Abstract

**Background:**

It has been shown that the pineal gland plays a fundamental role in mediating either the spiritual perception or the anticancer immunity by stimulating the endogenous production of anticancer cytokine interleukin (IL)-2.

**Objective:**

The present study was performed to evaluate the impact of a spiritual approach consisting of Kriya Yoga program alone or in association with melatonin (MLT) or low-dose IL-2 plus MLT on the survival time in a group of metastatic cancer patients with life expectancy less than 1 year.

**Materials and Methods:**

A case-control study was carried out in 240 patients (M/F: 146/94; median age: 62 years, range: 34-71, suffering from non-small-cell lung cancer or gastrointestinal tumors) who were subdivided into 6 groups of 40 patients, treated with supportive care alone as a control group, supportive care plus Yoga, MLT alone, MLT plus Yoga, inteleukin-2 plus MLT, or IL-2 plus MLT plus Yoga.

**Results:**

The best results in terms of increased survival time were obtained by the association between neuroimmunotherapy with MLT plus IL-2 and Yoga program (2 years), which was significantly longer with respect to that achieved by supportive care alone, Yoga alone, or IL-2 plus MLT alone (1 year).

**Conclusions:**

This study would suggest that a spiritual therapeutic approach may improve the survival time of untreatable metastatic solid tumor patients.

## INTRODUCTION

The recent advances in the Psychoneuroendocrino-immunology have demonstrated that not only the metabolic processes, but also the emotions and consciousness states, including psychedelic expansion of mind, are chemically mediated. The psychoneurobiochemistry is the link between physical existence and psychospiritual life.[1–3] Several psychoneuroendocrine and immune alterations have been demonstrated in cancer patients:[1] with regard to the immune cell alterations, decreased number and activity of natural killer cells, decreased number of T helper-1 (CD4) lymphocytes, decreased number and activity of dendritic cells and lymphocytopenia; with regard to the alterations of cytokine secretions, low levels of interleukin (IL)-2, high levels of IL-6, and high levels of IL-10; with regard to the neuroendocrine alterations, progressive decline in nocturnal production of the pineal hormone melatonin (MLT), enhanced brain opioid tone, decreased brain dopaminergic tone, paradoxical responses of the pituitary hormones, hyperprolactinemia, probable hypofunction of brain cannabinergic system. Cancer progression is characterized and determined by a great variety of both immune and psychoneuroendocrine alterations, which may be documented on a simple venous blood sample of patients and which consist of progressive decline in the absolute number of circulating lymphocytes, T lymphocytes, T-helper 1 lymphocytes, natural killer cells, and dendritic cells; progressive decline in IL-2 levels; increase in IL-l0 and IL-6 concentrations;[1,4,5] progressive decline in the endocrine function of the pineal gland; and increase in brain opioid activity.[1] In particular, the progressive decrease in the nocturnal production of the pineal hormone MLT,[1,6] with a following disappearance of its physiological light/dark circadian rhythm, would constitute the most frequent cancer progression-related endocrine disorder. Therefore, on the basis of the fact that cancer progression is associated with several neuroimmune disorders, the knowledge of the psychoneuroimmune physiopathology of cancer patients is fundamental to elaborate an interpretation of the neoplastic disease. In particular, because of the neurochemical mediation of the psychospiritual life and the evidence of psychoneuroendocrine alterations in cancer patients, there is no real psycho-oncology without the psychoneuroimmune knowledge. In fact, previous clinical studies have already demonstrated that the diminished capacity of feeling pleasure, which represents association with pain as one of the most frequent cancer-related symptoms, is determined by an altered dopaminergic function[7] which plays an essential role in the perception of pleasure,[1] including the sexual one. Moreover, it has been documented that the suppression of the sexual interest in cancer patients is constantly associated with an inhibition of the spiritual expression, by confirming the already demonstrated evidence of a similar neurobiochemistry of sexual and spiritual profiles,[1] even though the cultural tradition of western countries has generally separated sexuality from spirituality. In fact, previous clinical studies have already demonstrated the therapeutic efficacy of neuroimmunotherapy with subcutaneous (SC) low-dose IL-2 plus MLT in untreatable metastatic cancer patients, for whom no other standard effective therapy was available.[6] Moreover, in addition to the proposal of a spiritual therapeutic method, it is important to identify some criteria to recognize the presence of a real status of faith in patients. To explain the potential anticancer effect of faith and spiritual meditation, one of the major mechanisms would be represented by the activation of the pineal gland, whose antiproliferative and immunostimulating role has been well documented.[1] In fact, the pineal gland is activated by darkness and inhibited by the light.[1,3] Within the great variety of spiritual methods proposed by the religious traditions, a relevant importance has to be recognized to Yoga, since its rationale is scientifically founded on the reciprocal relation between respiration and emotional states.[8] Moreover, within the several types of Yoga[9] (Hata, Raja, Bakti, Karma, Jnana, Tantra, and Kriya), Kriya Yoga could be particularly appropriate for advanced cancer patients, because of no requirement of particular positions of the body, which would be difficult for patients with advanced disease, and of its ability to control not only the mental status through a conscious regulation of the physical respiration, but also to amplify the sentiments and the feeling of Love in the heart. On these bases, a study was performed to evaluate the influence of a Kriya Yoga program in a group of metastatic cancer patients who did not respond to previous anticancer conventional therapies and who were treated with supportive care alone, MLT alone,[10] low-dose IL-2 alone,[11] or IL-2 plus MLT.[12].

## MATERIALS AND METHODS

### Subjects

The study included 240 untreatable metastatic solid tumor patients (M/F: 146/94; median age: 62 years, range: 34-71), suffering from non-small-cell lung cancer (NSCLC) or gastrointestinal tumors. The study was approved by the Regional Ethics Committee. The study was explained to each person and they all signed an informed consent.

Eligibility criteria were as follows: histologically proven metastatic NSCLC or gastrointestinal tumor, measurable lesions, lack of response to previous conventional anticancer therapies, no double tumor, no brain metastases, no concomitant chronic therapy with corticosteroids or opioids because of their immunosuppressive activity on the anticancer immunity, and life expectancy lower than 1 year. Since the acceptance of the spiritual therapeutic program was spontaneous and free, it was not possible to realize a randomized study, but only a case-control study, by choosing control patients on the basis of the clinical characteristics of those patients who accepted to undergo a spiritual experience in association with supportive care alone, or the pineal hormone MLT plus low-dose IL-2. Patients were subdivided into 6 groups of 40 patients per group on the basis of the therapeutic approach, consisting of supportive care alone, supportive care plus Yoga, MLT alone, MLT plus Yoga, low-dose IL-2 plus MLT, or IL-2 plus MLT plus Yoga.

### Setting

The study was conducted between June 2008 and December 2010. Forty consecutive patients who did not respond to the line chemotherapy but only supportive care and who were admitted at Oncologist Department of the San Gerardo Hospital of Monza were recruited.

### Intervention

#### Medication schedule

According to the schedules of therapy previously described,[6,13] MLT was given orally at the pharmacological doses of 20 mg/day in the dark period of the day every day, corresponding to the daily period during which its endogenous production is maximal in physiological conditions.

IL-2 was injected SC at 3 million IU/day, 6 days/week for 4 consecutive weeks, corresponding to one complete immunotherapeutic cycle. In nonprogressing patients, a second cycle was planned after a 21-day rest period; then patients underwent a maintenance therapy consisting of 6 days of IL-2 treatment every month.

IL-2 and MLT therapy was continued without interruption until disease progression, as assessed by radiological examinations performed every 3 months.

### Kriya Yoga

The spiritual program of therapy consisted of Kriya Yoga in association with a meditation on biblical psalms, which were specific for each histotype of tumor. Kriya Yoga program was realized in a day-hospital regimen and it consisted of 2 hours of teaching at every 15-day intervals. Obviously, patients were invited to follow Yoga meditation for at least 15 minutes every day, early in the morning or before sleeping. Finally, patients who underwent a spiritual program were educated to live each day with joy (a glass of wine, a promenade in the nature, a sexual relation) as a spiritual experience in the status of thanksgiving to the life. The end-point of the study was the evaluation of the survival time. The clinical characteristics of patients are reported in [Table 1].

**Table 1:** Clinical characteristics of patients

	Total	SC	SCY	SCMLT	SCMLTY	SCIL2MLT	SCIL2MLTY
Numbers	240	40	40	40	40	40	40
Gender							
Males	146	24	25	24	25	24	24
Female	94	16	15	16	15	16	16
Age							
Range	34-71	40-70	34-63	37-69	35-68	35-70	34-71
Median	60	45	47	48	53	51	52
Tumor histotype							
NSCLC	175	30	28	29	30	28	30
Gi	65	10	11	12	11	11	10

SC – Supportive care, SCY – Supportive care plus Yoga, MLT – Melatonin, MLTY – Melatonin plus Yoga, IL2 MLT – Interleukin–2 plus melatonin, IL2MLTY – Interleukin– 2 plus Melatonin plus Yoga), NSCLC – Non–small–cell lung cancer

### Supportive care

Supportive care provides relief from pain and other distressing symptoms. We used narcotic drugs, including morphine, duragesic, and oxycodone.

### Measurements

The clinical response (disease status, tumor regression) was assessed according to WHO criteria and patients were evaluated radiologically. The subjective well-being and anxiety were evaluated by a clinical interview conducted by a psychologist. For the survival time, the log-rank test and Kaplan-Meier method were used.

### Statistical analysis

Data were statistically analyzed by the chi-square test for the clinical response, as assessed by WHO criteria, and by the log-rank test and Kaplan-Meier method for the survival time.

The results were considered to be statistically significant for *P*<0.05; α and β were 5% and 95%, respectively. Proportions were compared by using the chi-square test.

## RESULTS

### Well-being

The application of Yoga improved the subjective well-being in most patients who referred a relief of anxiety, serenity, relaxation, and a progressive increase in their consciousness and interpretation of the spiritual significance of disease.

### Tumor regression

No complete response was observed. A partial response (PR) was achieved in none of the patients treated with supportive care alone, supportive care plus Yoga, or MLT alone, in 1/40 (3%) patients treated with MLT plus Yoga, in 5/40 (13%) patients treated with IL-2 plus MLT, and in 8/40 (20%) patients treated with IL-2 plus MLT plus Yoga. A stable disease (SD) occurred in 2/40 (5%) patients treated with supportive care alone, in 5/40 (13%) patients treated with supportive care plus Yoga, in 9/40 (22%) patients who received MLT alone, in 11/40 (28%) patients treated with MLT plus Yoga, in 17/40 (43%) patients treated with IL-2 plus MLT alone, and in 24/40 (60%) patients who underwent a complete bioimmunospiritual program with IL-2 plus MLT and Yoga. Therefore, the percent of disease-control (PR+SD) was 32/40 (80%) in patients treated with IL-2, MLT, and Yoga, and it was significantly higher with respect to that achieved in patients treated with IL-2 plus MLT alone (22/40 [55%], *P*<0.05), in patients treated with MLT plus Yoga (12/40 [30%], *P*<0.025), in patients treated with MLT alone (9/40 [22%], *P*<0.01), Yoga plus supportive care (5/40 [13%], *P*<0.005), or supportive care alone (2/40 [5%], *P*<0.01).

### Survival

None of the patients treated with supportive care alone or supportive care plus Yoga was alive at 2 years, whereas a 2-year survival was obtained in 1/40 (3%) patient of MLT group, in 3/40 (8%) patients treated with MLT plus Yoga, in 6/40 (15%) patients treated with inteleukin-2 plus MLT alone, and in 9/40 (25%) patients treated with IL-2 and MLT plus Yoga.

The survival obtained in patients who underwent IL-2 and MLT plus Yoga program was significantly higher with respect to that observed in the other groups of patients (*P*<0.001 vs supportive care alone, *P*<0.005 vs supportive care plus Yoga or MLT alone, *P*<0.01 vs MLT plus Yoga, and *P*<0.05 *vs* IL-2 plus MLT).

The survival time observed in patients treated with supportive care plus Yoga was longer than that found in those treated with only supportive care, without, however, statistically significant differences. The survival times observed in the different groups of patients are reported in [Table 2].

**Table 2:** Percent of survival at the end of 1 and 2 years in the different groups

Groups	Survival time	
	1 Year	2 Years
SC	1/40 (3)	0
SCY	2/40 (5)	0
SCMLT	10/40 (25)	1/40 (3)
SCMLTY	15/40 (38)	3/40 (8)
SCIL2MLT	21/40 (53)	6/40 (15)**
SCIL2MLTY	29/40 (72)	9/40 (25)*

* *P*<0.001 vs SC; *P*<0.005 vs SCMLT, vs SCYOGA; *P*<0.01 vs SCMLTY; *P*<0.05 vs SCIL2MLT, SC – Supportive care, SCY – Supportive care plus yoga), MLT – Melatonin, MLTY – Melatonin plus yoga, IL2 MLT – Interleukin-2 plus melatonin, IL2MLTY – Interleukin–2 plus Melatonin plus Yoga, Figures in parentheses are in percentage

**Figure 1:**
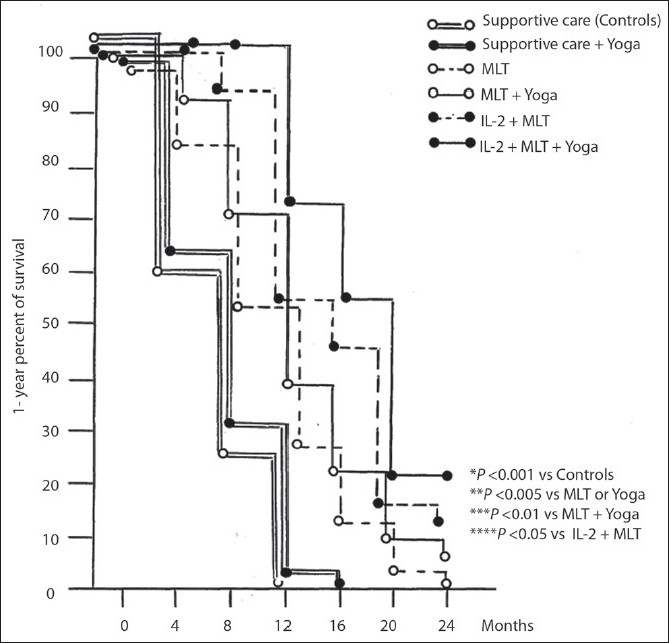
1-year survival curves achieved in metastatic cancer patients who failed to respond to standard anticancer therapies in relation to the type of therapeutic spiritual program

The Figure 1 shows the 1-years survival curves achieved in metastatic cancer patients, who failed to respond to standard anticancer therapies, in relation to the type of therapeutic spiritual program.

The clinical response achieved in the different groups of patients is shown in [Table 3].

**Table 3:** Tumor response according to WHO criteria in 240 metastatic cancer patients, who failed to respond to standard anticancer therapies, in relation to the type of therapeutic spiritual program

Therapeutic	Clinical response**					
approach	CR	PR	RR(CR+PR)	SD	DC(CR+PR+SD)	PD
SC	0	0	0 (0)	2	2 (5)	38
SCY	0	0	0 (0)	5	5 (13)	35
SCMLT	0	0	0 (0)	9	9 (22)	31
SCMLTY	0	1	1 (3)	11	12 (30)	28
SCIL2MLT	0	5	5 (13)	17	22 (55)	18
SCIL2MLTY	0	8	8 (20)	24	32(80)*	8

* *P*<0.05 vs SCIL2MLT; *P*<0.025 *vs* SCMLTY; *P*<0.01 *vs* SCMLT; *P*<0.005 *vs* SCY; *P*<0.001 vs SC

** CR– Complete response; PR – Partial response; SD – Stable disease; DC – Disease control; PD – progressive disease, Figures in parentheses are in percentage

## DISCUSSION

The results of the present study suggests that either a spiritual approach, such as a Yoga program, or a neuroimmunotherapeutic strategy, carried out to correct the major cancer-related neuroendocrine or immune deficiencies, may prolong the survival time in metastatic solid tumor patients, who did not respond to previous conventional anticancer therapies and for whom no other standard treatment was available.

At present, it is known that the anticancer immunity is fundamentally an IL-2-dependent phenomenon,[14,4] which is amplified by IL-12 and inhibited by IL-l0 and IL-6. IL-2, released from T helper-1 lymphocytes, induces the evolution of natural killer cells into LAK cells,[14] which are able to exert an antigen-independent cancer cell destruction. IL-12, produced by dentritic cells (DC), stimulates T cytotoxic lymphocytes,[11] which mediate an antigen-dependent cytotoxicity. IL-6, released from macrophages, suppresses IL-2-induced generation of LAK cells.[15] Finally, IL-10, released from T-helper-2 (TH2) lymphocytes, may inhibit the production of both IL-2 and IL-12.[15] Even though all neuropeptides and neurohormones may have potentiality to exert immunomodulating effects,[1–3] it is possible to recognize two fundamental psychoneuroendocrine systems in the brain responsible for the psychospiritual control of the immune response.[1] The first system consists of the functional unity of noradrenergic pathways, opioid system, and pituitary gland. This network mediates stress, anxiety, pain, and depression which is related to the unconscious life. Its activation induces immune suppression through a stimulation of T-helper 2 lymphocytes followed by enhanced release of IL-10, which inhibits IL-2 and IL-12 secretion.[5] The second system, represented by the functional axis of pineal gland, gabaergic-type A pathways, and cannabinergic system, is related to the perception of pleasure and to the spiritual expansion of consciousness state.[1,16] Its activation increases immunostimulation, including the stimulation of the antitumor immunity, by activating T-helper 1 lymphocytes and dendritic cells, followed by enhanced production of IL-2 and IL-12, respectively.[17] Within the psychoneuroimmune system, a fundamental role is played by the pineal gland, which would represent a central regulator of cytokine network.[18] The pineal gland exerts an essential role in mediating the relation either between the universal life and the single living organism, or between the psychospiritual condition and the immunological functions.[6] Moreover, the pineal gland may release various indole hormones provided not only by immunostimulating effect but also by a direct antitumor antiproliferative action,[19] the most investigated of them is represented by MLT.[6] There are several studies in literature that have used cancer immunotherapy with IL-2, showing good results in treating kidney cancer. The studies on MLT in patients with different histotypes tumor have led to good results. To date, however, studies which are combined with immunotherapy and treatment with MLT and spiritual approach are lacking. The recent advances in the psycho-oncological and psychoneuroimmunological investigations of cancer patients have allowed the rediscovery of the importance of spiritual faith in influencing the clinical course of neoplastic disease, not only in terms of supportive care but also as a potential prognostic variable. This statement is justified by recent studies suggesting that spiritual support may allow an increase in the survival time of advanced cancer patients. However, little is known regarding how Yoga may influence the clinical course of neoplastic disease, even though some preliminary studies would suggest that a high spiritual status may counteract cancer growth by stimulating the anticancer immune responses, which are mainly mediated by lymphocytes.

Yoga applied to cancer disease can improve the quality of life and subjective well-being of the patient. There are currently no studies that combine spiritual techniques and immunotherapy in cancer care.

Moreover, this study shows that the best results in terms of both objective tumor regression and increased survival time may be achieved when the spiritual approach was associated with a neuroendocrinoimmune chemical therapeutic strategy, such as low-dose IL-2 plus the pineal hormone MLT, carried out to abrogate the main neuroimmune deficiencies occurring in metastatic cancer patients. This finding is not surprising by taking into consideration that no spiritual status may be expressed independently of the psychoneurobiochemical status. It has been proven to be compromised with cancer progression, which has appeared to be associated with molecular deficiencies exactly involving those neurobiochemical systems responsible for the chemical mediation of both pleasure and spiritual feeling. Therefore, on the basis of the psychoneuroimmune knowledge available up to now and of the results of this study, each type of spiritual approach, which excludes a concomitant neuroimmunotherapeutic approach to restore the neuroimmune biochemistry of the status of health, would have to be considered as an inadequate strategy. This is because cancer progression is a systemic disease, characterized by a progressive molecular deficiency involving those systems responsible for the maintenance of the biopsychospiritual unity of human beings, such as the immune system and the pineal gland.

It has been shown that the concomitant administration of IL-12 may amplify the anticancer immunity induced by IL-2 alone.[20]

In the same way, pineal indoles other than MLT[21] and the endogenous cannabinoid agonist anandamide may further enhance the anticancer biological response of patients.[22] This statement is justified by the fact that cancer-related immunoneuroendocrine molecular deficiencies are not a simple epiphenomenon associated with tumor dissemination, since their correction through an exogenous administration of the most important molecules responsible for the generation of an effective antitumor neuroimmune response, namely IL-2 and pineal indoles, may induce a control of the neoplastic growth.[23]

Because patients of the different tumor histotypes are not evaluated in this study, further clinical investigations in a greater number of patients for each single histotype will be required to better define the impact of a spiritual approach in untreatable cancer patients.

Therefore, these alternative anticancer biotherapies might be successfully introduced with benefits within the possible cures of the medical oncology, and commonly used in the treatment of human advanced neoplasms.

In any case, the results of this study may be considered as the basis of future clinical researches. In fact, more promising results could be achieved through a more intensive program of Yoga and other neuroimmunotherapeutic combinations.

The best spiritual approach to be recommended to untreatable metastatic cancer patients is that associated with a concomitant neuroimmunotherapeutic strategy, beyond the separation between psychospiritual status and neuroimmune biochemistry, as well as beyond the opposition between material pleasure and spiritual feeling, in an attempt to transform the material life in a living liturgy.
